# Preoperative high-sensitivity C-reactive protein-to-albumin ratio predicts arteriovenous fistula failure in hemodialysis patients: a retrospective cohort study with landmark analysis

**DOI:** 10.3389/fsurg.2026.1922562

**Published:** 2026-09-09

**Authors:** Shanshan Huo, Peng Shu, Zhuping Wen, Xiaodi Ma, Shuang Wang, Fang Xu

**Affiliations:** Department of Nephrology, The Central Hospital of Wuhan, Tongji Medical College, Huazhong University of Science and Technology, Wuhan, Hubei, China

**Keywords:** albumin, arteriovenous fistula, C-reactive protein, hemodialysis, inflammation

## Abstract

**Background:**

The arteriovenous fistula (AVF) is the preferred vascular access for hemodialysis, yet up to 50% of AVFs fail within the first year due to stenosis or thrombosis. The high-sensitivity C-reactive protein to albumin ratio (HRR) integrates inflammation and nutritional status, but its association with AVF failure has not been systematically evaluated.

**Methods:**

This retrospective cohort study included 279 patients who underwent first-time AVF creation. HRR was calculated as hsCRP (mg/L) divided by albumin (g/L). The primary outcome was AVF failure (stenosis ≥50% or thrombosis requiring intervention). Multivariate Cox regression adjusted for age, sex, BMI, diabetes, hypertension, smoking, and vessel diameter. Model performance was assessed using time-dependent ROC, calibration curve, and decision curve analysis (DCA).

**Results:**

During a median follow-up of 50 months, 141 patients (50.5%) developed AVF failure.In multivariate analysis, higher HRR was independently associated with increased AVF failure risk (HR per unit increase = 1.65, 95% CI: 1.33–2.05, *P* < 0.001), independent of vessel diameter (HR = 0.21, 95% CI: 0.08–0.57, *P* = 0.002). A landmark analysis stratified at 24 months demonstrated that the predictive effect of HRR was directionally consistent across early (HR = 1.60, 95% CI: 1.27–2.02) and late (HR = 1.47, 95% CI: 0.66–3.29) follow-up periods (*P* for interactio*n* = 0.766), although the late-phase estimate was imprecise due to the reduced sample size. The model demonstrated moderate discrimination at 24 months (AUC = 0.608, 95% CI: 0.519–0.701) and good calibration. DCA confirmed that incorporating HRR provided positive net clinical benefit across clinically relevant threshold probabilities. The proportional hazards assumption was satisfied (global *P* = 0.97). Sensitivity analysis using multiple imputation yielded consistent results.

**Conclusions:**

Preoperative HRR is an independent predictor of AVF failure. Although the model demonstrated weak to moderate discrimination (24-month AUC = 0.608), decision curve analysis confirmed a positive net clinical benefit, supporting its potential utility in preoperative risk stratification. This simple, widely available laboratory-based marker may assist clinicians in identifying high-risk patients and individualizing surveillance intensity, though external validation is warranted.

## 1 Introduction

Chronic kidney disease (CKD) has emerged as a major public health challenge worldwide, with an estimated global prevalence of 14.3% and a disproportionate burden in low- and middle-income countries (1). In China, an estimated 152 million adults live with chronic kidney disease (CKD; 95% uncertainty interval, 142–164 million), and a growing share of these individuals progress to end-stage kidney disease (ESKD) requiring long-term renal replacement therapy (1). Among the available modalities, maintenance hemodialysis remains the predominant treatment, serving more than 80% of ESKD patients in China. The arteriovenous fistula (AVF) is universally recommended as the preferred vascular access by the Kidney Disease Outcomes Quality Initiative (KDOQI) and the Chinese Vascular Access Consensus Guidelines due to its superior long-term patency, lower infection rates, and reduced healthcare costs compared to arteriovenous grafts or central venous catheters (2, 3). However, the clinical success of AVF depends not only on surgical technique but also on patient-specific factors that influence vascular adaptation and remodeling.

Despite its established advantages, AVF failure remains a major clinical problem. Depending on the definition and follow-up duration, primary failure rates range from 20% to 50%, and up to 60% of AVFs fail to mature adequately for hemodialysis use (4). The predominant mechanism underlying AVF failure is neointimal hyperplasia—a pathologic process characterized by smooth muscle cell proliferation, migration, and extracellular matrix deposition—which ultimately leads to progressive luminal stenosis and, in many cases, thrombosis (5, 6). This process is driven by a complex interplay between local hemodynamic forces, surgical injury, and systemic patient factors. Multiple risk factors have been consistently identified, including advanced age, female sex, diabetes mellitus, hypertension, and suboptimal vessel diameter, with the latter being a particularly strong predictor of maturation failure (7–9). More recently, accumulating evidence has highlighted the role of systemic inflammation and poor nutritional status in the pathogenesis of vascular dysfunction, suggesting that these modifiable factors may represent promising therapeutic targets and prognostic markers (10, 11). Nevertheless, existing risk stratification tools remain limited, and simple, cost-effective preoperative markers capable of identifying high-risk patients are still lacking in routine clinical practice.

Inflammation and malnutrition are increasingly recognized as key determinants of AVF outcome. Elevated high-sensitivity C-reactive protein (hsCRP), a sensitive biomarker of systemic inflammation, has been shown to predict cardiovascular events and all-cause mortality in patients with ESKD (12). Mechanistically, hsCRP promotes endothelial dysfunction, oxidative stress, and smooth muscle cell proliferation—all processes that directly contribute to neointimal hyperplasia and subsequent AVF stenosis (13). Concurrently, hypoalbuminemia, reflecting poor nutritional reserve, has been independently associated with impaired vascular healing and increased infection risk following vascular access surgery (14). The prognostic nutritional index (PNI) and other nutrition-based scores have demonstrated predictive value for adverse outcomes in dialysis patients (15), further supporting the biological link between nutritional status and vascular health. More importantly, the hsCRP-to-albumin ratio (HRR)—a composite index that integrates both inflammatory and nutritional information—has emerged as a robust prognostic marker in diverse clinical settings, including cardiovascular disease, cancer, and sepsis (16, 17). However, despite the biological plausibility and the established value of HRR in other fields, its association with AVF failure has not been systematically investigated. Few studies have incorporated inflammatory-nutritional markers into AVF outcome prediction, and none, to our knowledge, have comprehensively validated HRR's predictive performance using contemporary model evaluation techniques.

Although recent studies have explored the association between inflammatory-nutritional markers and AVF outcomes, two important gaps remain. First, the predictive value of HRR specifically for clinically significant AVF failure (stenosis ≥50% or thrombosis requiring intervention)—as opposed to maturation failure—has not been rigorously evaluated with comprehensive model performance metrics including calibration and decision curve analysis. Second, whether the effect of HRR varies over time (i.e., early vs. late postoperative phases) remains unknown. To address these gaps, we conducted this retrospective cohort study with three objectives: (1) to evaluate the independent association between preoperative HRR and AVF failure after adjusting for established risk factors including vessel diameter; (2) to assess the model's discrimination, calibration, and clinical net benefit following TRIPOD (Transparent Reporting of a Multivariable Prediction Model for Individual Prognosis or Diagnosis) statement for prognostic model development and validation (18).

(3) To perform a landmark analysis at 24 months to examine the temporal stability of HRR's predictive effect. We hypothesized that HRR would be independently associated with AVF failure and that its effect would remain consistent over time.

Therefore, the aim of this study was to evaluate the association between preoperative HRR and AVF failure in a Chinese hemodialysis cohort with long-term follow-up, and to comprehensively assess the predictive performance (discrimination, calibration, and clinical net benefit) of a model incorporating HRR and guideline-recommended risk factors. We hypothesized that higher HRR would be independently associated with increased AVF failure risk, and that the HRR-based model would demonstrate acceptable performance and clinical utility. Additionally, we performed a landmark analysis at 24 months to examine whether the predictive effect of HRR varies over time, an aspect not previously investigated. Our findings may complement existing risk assessment strategies by providing a simple, widely available laboratory-based parameter for preoperative stratification in vascular access management.

## 2 Methods

### 2.1 Study design and population

This retrospective cohort study was conducted at The Central Hospital of Wuhan and included consecutive patients who underwent first-time AVF creation between January 2022 and December 2025. Inclusion criteria were: (1) age ≥ 18 years; (2) first AVF creation in the upper extremity; (3) complete preoperative laboratory data available; and (4) at least one follow-up visit after AVF creation. Exclusion criteria were: (1) previous AVF or arteriovenous graft in the same limb; (2) active infection or malignancy at the time of surgery; (3) incomplete follow-up data; and (4) loss to follow-up within 30 days postoperatively. The study was approved by the Institutional Review Board of The Central Hospital of Wuhan (No.WHZXKYL2024-115). Written informed consent was waived due to the retrospective nature of the analysis.

### 2.2 Data collection

Baseline demographic and clinical characteristics were extracted from electronic medical records, including age, sex, body mass index (BMI), smoking status, and comorbidities (hypertension, diabetes mellitus). Preoperative laboratory measurements were obtained within 30 days prior to AVF creation, including high-sensitivity C-reactive protein (hsCRP), serum albumin, and complete blood count. Vessel diameter was measured by preoperative duplex ultrasound at the planned anastomotic site. Both the cephalic vein and radial artery diameters were recorded; the venous diameter (cephalic vein) was used in the regression models, as it is the primary anatomical determinant of AVF maturation according to KDOQI guidelines. All measurements were performed by experienced vascular sonographers according to standard institutional protocols.

Variables for multivariate models were prespecified based on clinical importance and prior evidence rather than data-driven selection. HRR was chosen as the primary exposure because it integrates inflammation (hsCRP) and nutritional status (albumin), both of which have been independently associated with AVF failure in previous studies (19). Covariates (age, sex, BMI, diabetes, hypertension, smoking, and vessel diameter) were selected based on KDOQI guidelines and established risk factors for AVF failure (3).

### 2.3 HRR calculation

HRR was calculated as the ratio of high-sensitivity C-reactive protein (mg/L) to serum albumin (g/L): HRR = hsCRP (mg/L)/albumin (g/L). For patients with albumin measured in different units, values were converted accordingly before calculation.

### 2.4 Outcome definition

The primary outcome was AVF failure, defined as a composite of radiologically confirmed stenosis (≥50% luminal narrowing) or thrombosis requiring intervention (percutaneous transluminal angioplasty, surgical revision, or creation of a new access). Outcome events were adjudicated by two independent clinicians who were blinded to the HRR values at the time of outcome assessment. Inter-observer agreement was assessed, with any disagreements resolved by consensus.

### 2.5 Follow-up

Patients were followed from the date of AVF creation until the occurrence of AVF failure, death, loss to follow-up, or the study termination date (December 31, 2025), whichever occurred first. Standardized follow-up visits were scheduled at 1, 3, 6, and 12 months postoperatively, and every 6 months thereafter. At each visit, clinical examination was performed and duplex ultrasound was routinely conducted to assess AVF patency, flow volume, and the presence of stenosis.

### 2.6 Statistical analysis

The required sample size was estimated based on the events per variable (EPV) principle. With 8 prespecified covariates in the multivariate model and 141 observed AVF failure events, the EPV was 141/8 ≈ 17.6, exceeding the commonly recommended minimum of 10 events per variable. This indicated that the sample size was adequate for the planned multivariable Cox regression analyses and provided sufficient statistical power to detect a clinically meaningful effect size. For the time-dependent ROC analysis, the sample size (279 patients, 141 events) allowed estimation of AUC values with acceptable precision, and 200 bootstrap resamples were performed to obtain stable 95% confidence intervals. The calibration curve and decision curve analysis were conducted descriptively without formal sample size calculation, as recommended for model performance evaluation in moderate-sized cohorts (20). Continuous variables were expressed as median with interquartile range (IQR) and compared using the Mann-Whitney U test. Categorical variables were presented as frequencies with percentages and compared using the chi-square test or Fisher’s exact test as appropriate.

Survival curves were estimated using the Kaplan-Meier method and compared using the log-rank test. Univariate and multivariate Cox proportional hazards regression models were used to estimate hazard ratios (HRs) and 95% confidence intervals (CIs) for the association between HRR and AVF failure. Variables were selected for multivariate models based on clinical importance and KDOQI guideline recommendations, including age, sex, BMI, diabetes, hypertension, smoking, and vessel diameter. The proportional hazards assumption was assessed using Schoenfeld residuals.

To comprehensively evaluate the predictive performance of the model, we conducted:

Time-dependent ROC analysis with AUC values at 12, 24, and 36 months, with 95% CIs estimated via bootstrap resampling (200 replicates);
Calibration curve at 12 months using the rms package; and
Decision curve analysis (DCA) at 24 months to assess clinical net benefit.
Median follow-up time was estimated using the reverse Kaplan-Meier method.

To assess whether the predictive effect of HRR varies over time, a landmark analysis stratified at 24 months post-surgery was performed. This time point was selected *a priori* to differentiate early failures (which may be predominantly influenced by surgical technique and acute anatomical factors) from late failures (which are more likely driven by progressive neointimal hyperplasia)^8,9^. Two separate Cox regression models were fitted: (1) an early-phase model including all patients, with events censored at 24 months; and (2) a late-phase model including only patients who survived event-free to 24 months, with follow-up time reset to zero at the landmark. To formally test for time-dependent effect modification, an interaction term between HRR and a time-period indicator (>24 months vs. ≤ 24 months) was included in the full Cox model.

### 2.7 Missing data handling

Missing data were present in several variables (hsCRP: 39.4%; BMI: 19.8%) (Supplementary Table S1). Complete-case analysis was used as the primary analytical approach. To assess the robustness of our findings, multiple imputation with chained equations (MICE) was performed using 5 imputed datasets, with the same covariates included in the imputation model. Results from complete-case and imputed analyses were compared as a sensitivity analysis.

### 2.8 Software and reproducibility

All statistical analyses were performed using R software version 4.4.2 (R Foundation for Statistical Computing, Vienna, Austria). Key packages included survival, survminer, timeROC, rms, dcurves, and mice. The complete analysis code and an anonymized version of the dataset are provided in the supplementary materials to ensure reproducibility. Statistical significance was set at *P* < 0.05 (two-sided).

## 3 Results

### 3.1 Patient disposition and baseline characteristics

**Figure 1:**
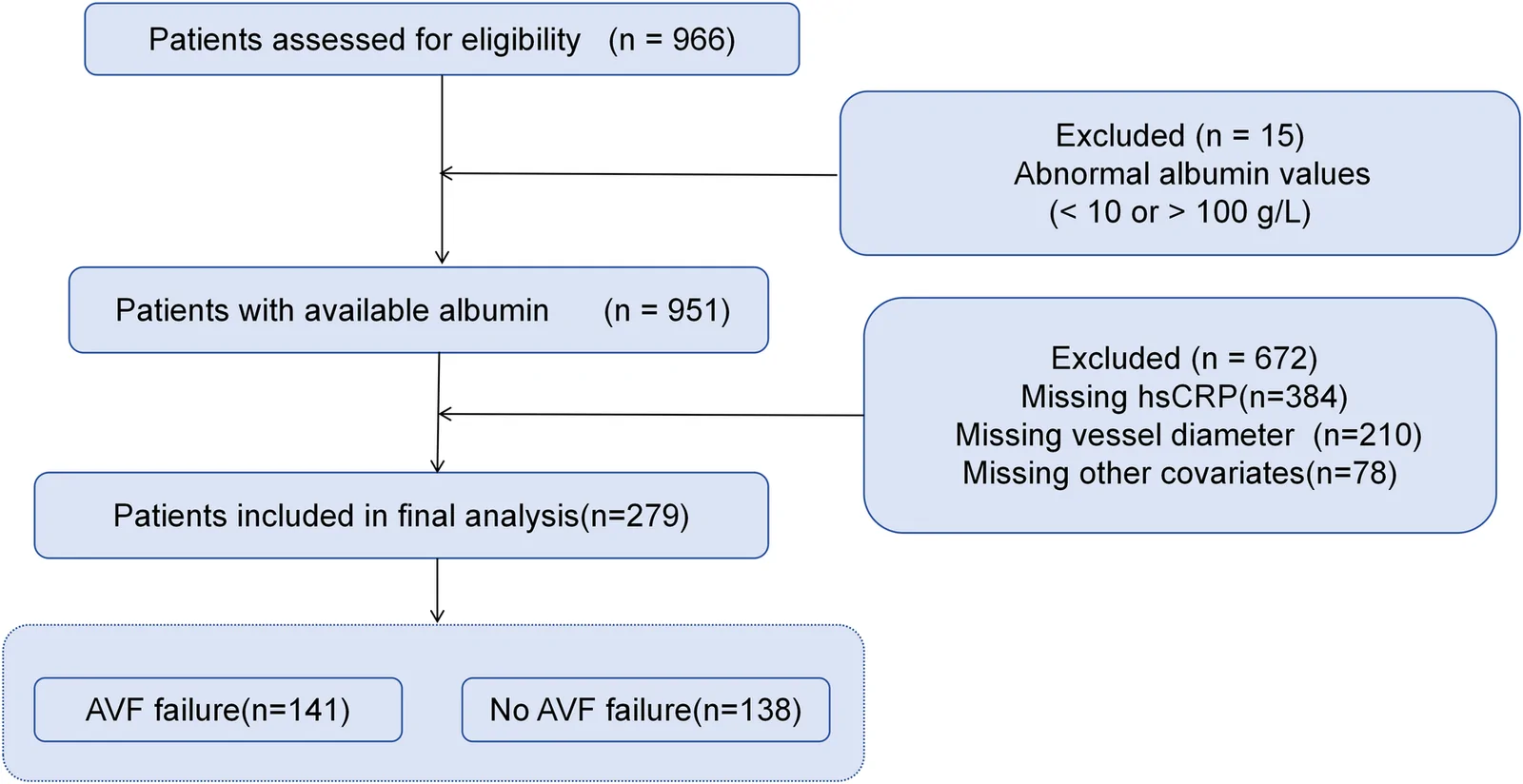
Study flow diagram. A total of 966 patients who underwent first-time AVF creation were initially screened. After excluding 15 patients with abnormal albumin values (<10 or >100 g/L) and 672 patients with missing key covariates (including hsCRP, vessel diameter, BMI, or follow-up data), 279 patients were included in the final analysis. Among these, 141 patients experienced AVF failure and 138 patients did not.

A total of 966 patients who underwent first-time AVF creation were initially screened. After excluding 15 patients with abnormal albumin values (<10 or >100 g/L) and 672 patients with missing key covariates (including hsCRP, vessel diameter, BMI, or follow-up data), 279 patients were included in the final analysis. The study flow diagram is presented in Figure 1.

Among the 279 included patients, 141 (50.5%) experienced AVF failure during follow-up; the remaining 138 patients were event-free and censored at study termination (December 31, 2025). The median follow-up time was 50 months. Of the 141 failure events, only 7 (5.0%) occurred within the first 6 months postoperatively, representing primary maturation failure, while the remaining 134 (95.0%) occurred after 6 months, representing late failure due to progressive stenosis or thrombosis. The cumulative failure rates at 12 and 24 months were 17.0% and 41.8%, respectively.

Baseline characteristics stratified by AVF failure status are summarized in Table 1. Patients with AVF failure had a significantly higher prevalence of diabetes (83.0% vs. 67.4%, *P* = 0.004) compared to those without failure. No statistically significant differences were observed between the two groups for age, BMI, HRR, vessel diameter, sex, smoking, or hypertension (all *P* > 0.05). Notably, vessel diameter showed a borderline trend toward smaller values in the failure group [median: 0.38 [IQR: 0.30–0.49] vs. 0.41 [IQR: 0.33–0.53], *P* = 0.070].

**Table 1:** Baseline characteristics stratified by AVF failure.

Variable	No AVF failure	AVF failure	P.value
Age (years)	62 (50.25–69)	62 (54–69)	0.203
BMI (kg/m^2^)	23.52 (22.02–25.95)	23.5 (20.6–25.1)	0.065
HRR	0.04 (0.01–0.15)	0.04 (0–0.14)	0.292
Vessel diameter (mm)	4.1 (3.3–5.3)	3.8 (3.0–4.9)	0.07
Male [*n* (%)]	96 (69.6%)	89 (63.1%)	0.312
Smoking [*n* (%)]	27 (19.6%)	28 (19.9%)	1
Hypertension [*n* (%)]	126 (91.3%)	135 (95.7%)	0.206
Diabetes [*n* (%)]	93 (67.4%)	117 (83%)	0.004

### 3.2 Univariate Cox regression analysis

Univariate Cox regression analysis was performed to identify factors associated with AVF failure. The results are presented in Table 2.

**Table 2:** Univariate Cox regression analysis for AVF failure.

Variable	HR	CI_lower	CI_upper	P
Age	1.004	1	1.008	0.0686
Gender	1.179	0.832	1.671	0.3556
BMI	1.001	0.973	1.029	0.9581
Smoking	1.125	0.74	1.708	0.5819
Hypertension	1.087	0.478	2.468	0.8429
Diabetes	1.426	0.917	2.218	0.1152
HRR	1.601	1.295	1.98	<0.001
Vessel Diameter	0.243	0.094	0.625	0.0034

HRR was significantly associated with an increased risk of AVF failure (HR = 1.60, 95% CI: 1.30–1.98, *P* < 0.001). Vessel diameter was also a significant predictor (HR = 0.24, 95% CI: 0.09–0.63, *P* = 0.003). Diabetes, age, sex, BMI, smoking, and hypertension did not reach statistical significance in the univariate analysis (all *P* > 0.05).

### 3.3 Multivariate Cox regression analysis

Multivariate Cox regression analysis was performed to evaluate the independent association between HRR and AVF failure after adjusting for prespecified covariates, including age, sex, BMI, diabetes, hypertension, smoking, and vessel diameter. The model included 8 covariates. With 141 events, the events per variable (EPV) was 17.6, exceeding the recommended minimum of 10, indicating adequate statistical power for the analysis.

**Figure 2:**
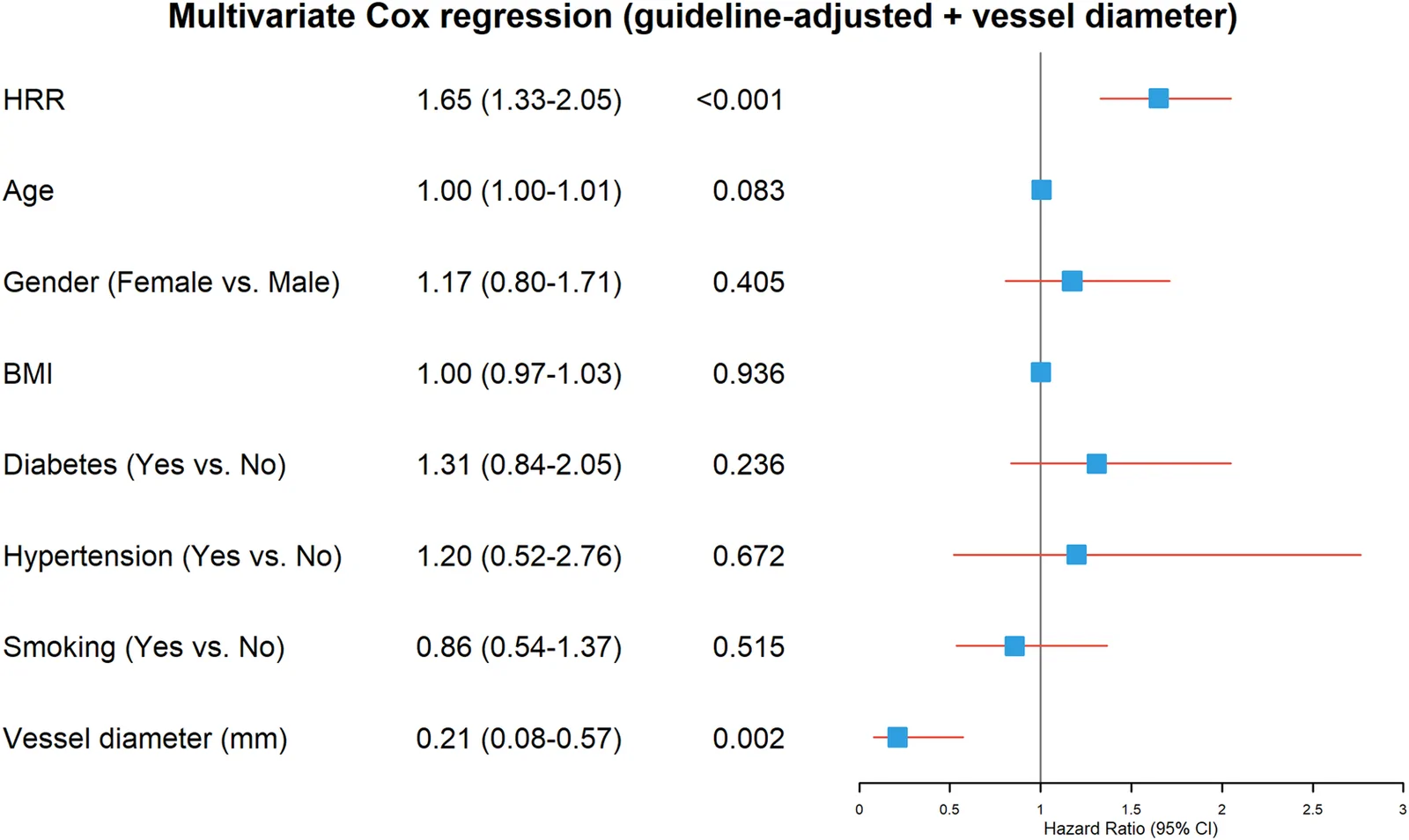
Forest plot of multivariate Cox regression analysis. Multivariate Cox regression model adjusted for age, sex, BMI, diabetes, hypertension, smoking, and vessel diameter. HRR: HR = 1.65 (95% CI: 1.33–2.05, *P* < 0.001); vessel diameter: HR = 0.21 (95% CI: 0.08–0.57, *P* = 0.002).

The results are presented in Table 3 and visualized in Figure 2.

**Table 3:** Multivariate Cox regression analysis for AVF failure.

Variable	HR	CI_lower	CI_upper	P
HRR	1.65	1.328	2.049	<0.001
Age	1.004	0.999	1.008	0.083
Gender (Female vs. Male)	1.174	0.805	1.711	0.4054
BMI	1.001	0.973	1.03	0.9364
Diabetes (Yes vs. No)	1.31	0.838	2.049	0.2365
Hypertension (Yes vs. No)	1.198	0.519	2.764	0.6719
Smoking (Yes vs. No)	0.856	0.537	1.365	0.5145
Vessel diameter (mm)	0.211	0.078	0.572	0.0022

HRR was strongly and independently associated with AVF failure (HR = 1.65, 95% CI: 1.33–2.05, *P* < 0.001), indicating that each unit increase in HRR was associated with a 65% higher risk of AVF failure.

Vessel diameter was also independently associated with AVF failure (HR = 0.21, 95% CI: 0.08–0.57, *P* = 0.002), indicating that each 1-mm increase in vessel diameter was associated with a 79% lower risk of AVF failure.

Among the other covariates, age showed a borderline association (HR = 1.004, 95% CI: 1.000–1.008, *P* = 0.083). Diabetes, hypertension, smoking, sex, and BMI did not reach statistical significance in the multivariate model (all *P* > 0.05).

The model demonstrated a C-index of 0.594 (SE = 0.027), indicating moderate discrimination. The likelihood ratio test (*χ*^2^ = 27.74, df = 8, *P* = 0.0005), Wald test (*P* = 0.00005), and score test (*P* = 0.00001) all confirmed that the model was statistically significant. The proportional hazards assumption was satisfied for all covariates (global *P* = 0.97).

### 3.4 Kaplan-meier survival analysis

**Figure 3:**
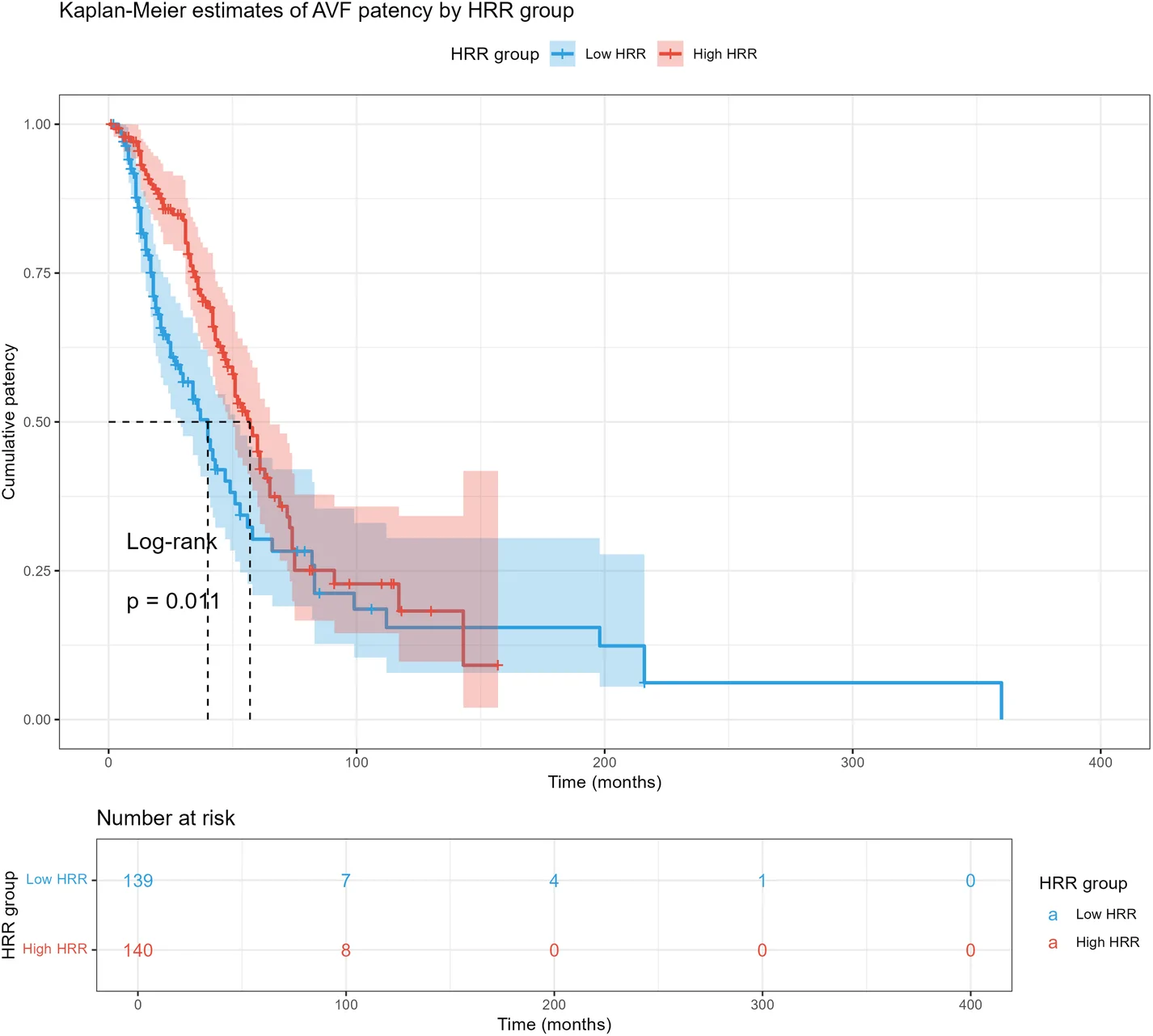
Kaplan-meier curves for AVF patency stratified by HRR group. Patients were divided into high and low HRR groups based on the median HRR value. The high HRR group showed significantly lower cumulative patency compared to the low HRR group (log-rank *P* = 0.011). The median patency time was 60 months (95% CI: 46–74) in the low HRR group and 34 months (95% CI: 24–44) in the high HRR group.

Kaplan-Meier curves stratified by HRR group (high vs. low, based on the median HRR value) are shown in Figure 3. Patients with high HRR had significantly lower cumulative AVF patency compared to those with low HRR (log-rank *P* = 0.011).The number of patients at risk at each time point is displayed in Supplementary Table S2.

In the low HRR group, the median AVF patency time was 60 months (95% CI: 46–74), while in the high HRR group it was 34 months (95% CI: 24–44).

### 3.5 Model performance evaluation

The predictive performance of the multivariate model was evaluated using time-dependent ROC analysis, calibration curve, and decision curve analysis (DCA).

**Figure 4:**
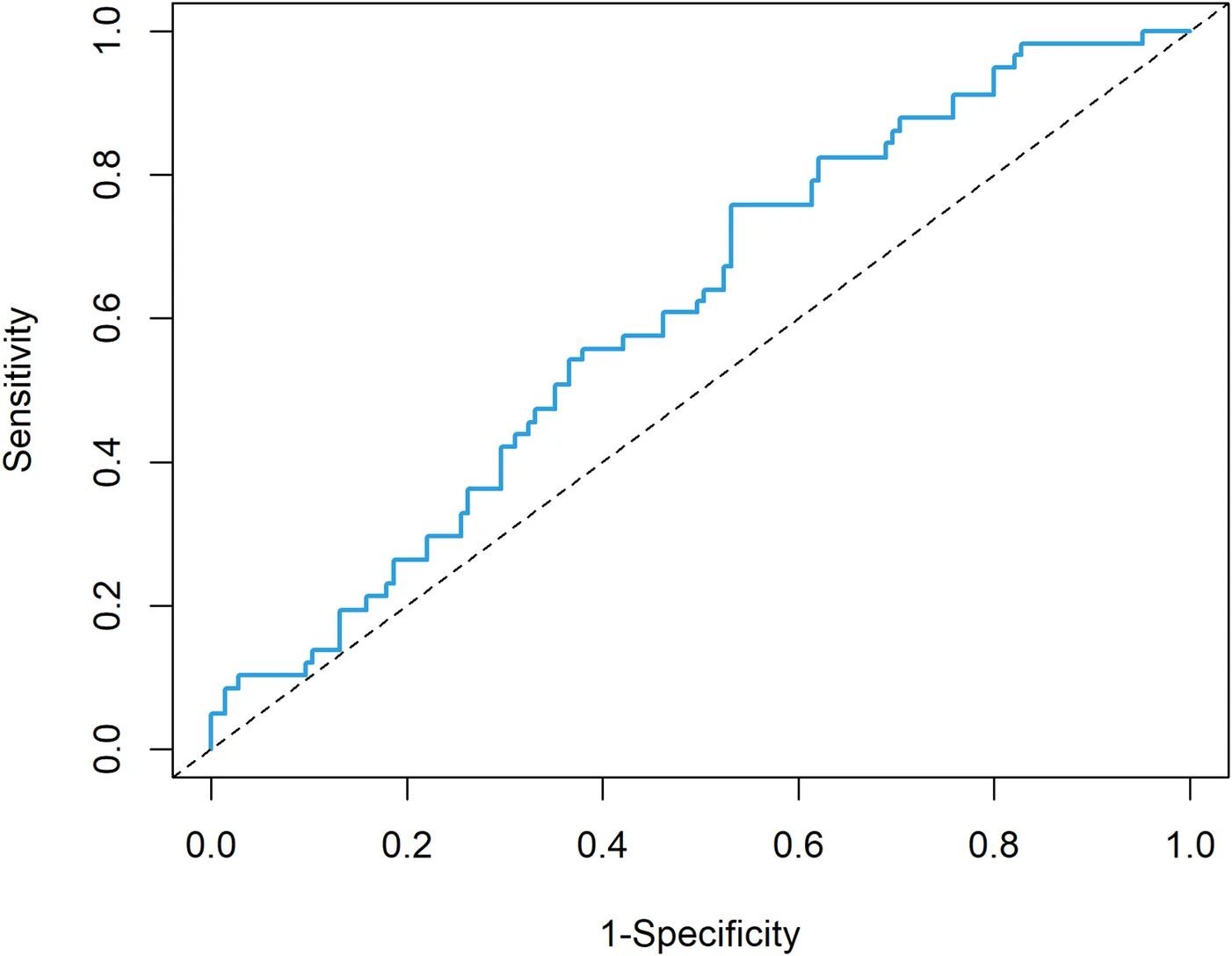
Time-dependent ROC curve at 24 months. The AUC was 0.608 (95% CI: 0.519–0.701), indicating moderate discrimination.

Time-dependent ROC analysis yielded AUC values of 0.533 (95% CI: 0.424–0.659) at 12 months, 0.608 (95% CI: 0.519–0.701) at 24 months, and 0.625 (95% CI: 0.537–0.717) at 36 months. The 24-month ROC curve is presented in Figure 4.

**Figure 5:**
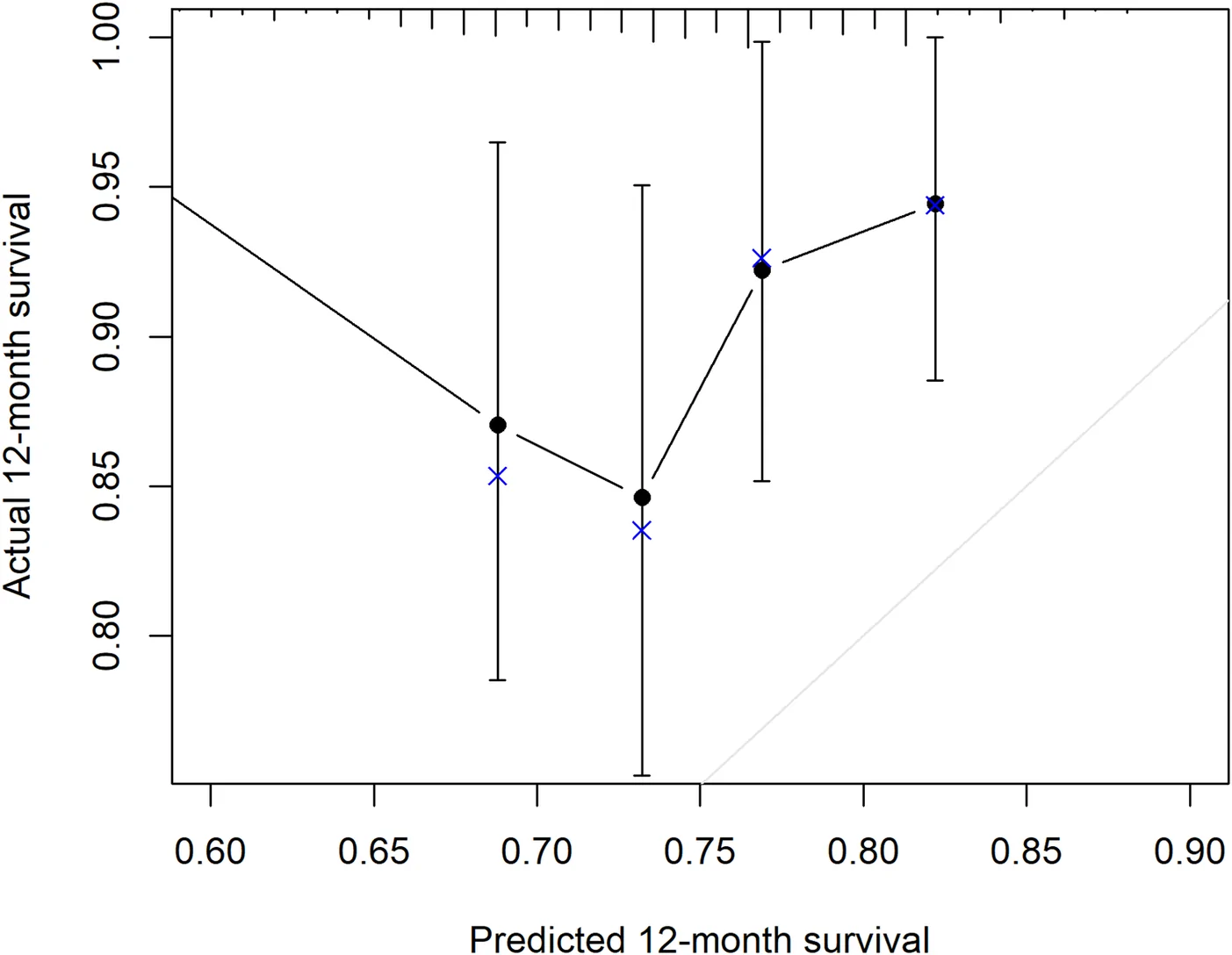
Calibration curve at 12 months. The curve shows acceptable agreement between predicted and observed survival probabilities, indicating that the model is reasonably well-calibrated.

The calibration curve at 12 months showed good agreement between predicted and observed survival probabilities (Figure 5), indicating that the model was well-calibrated.

**Figure 6:**
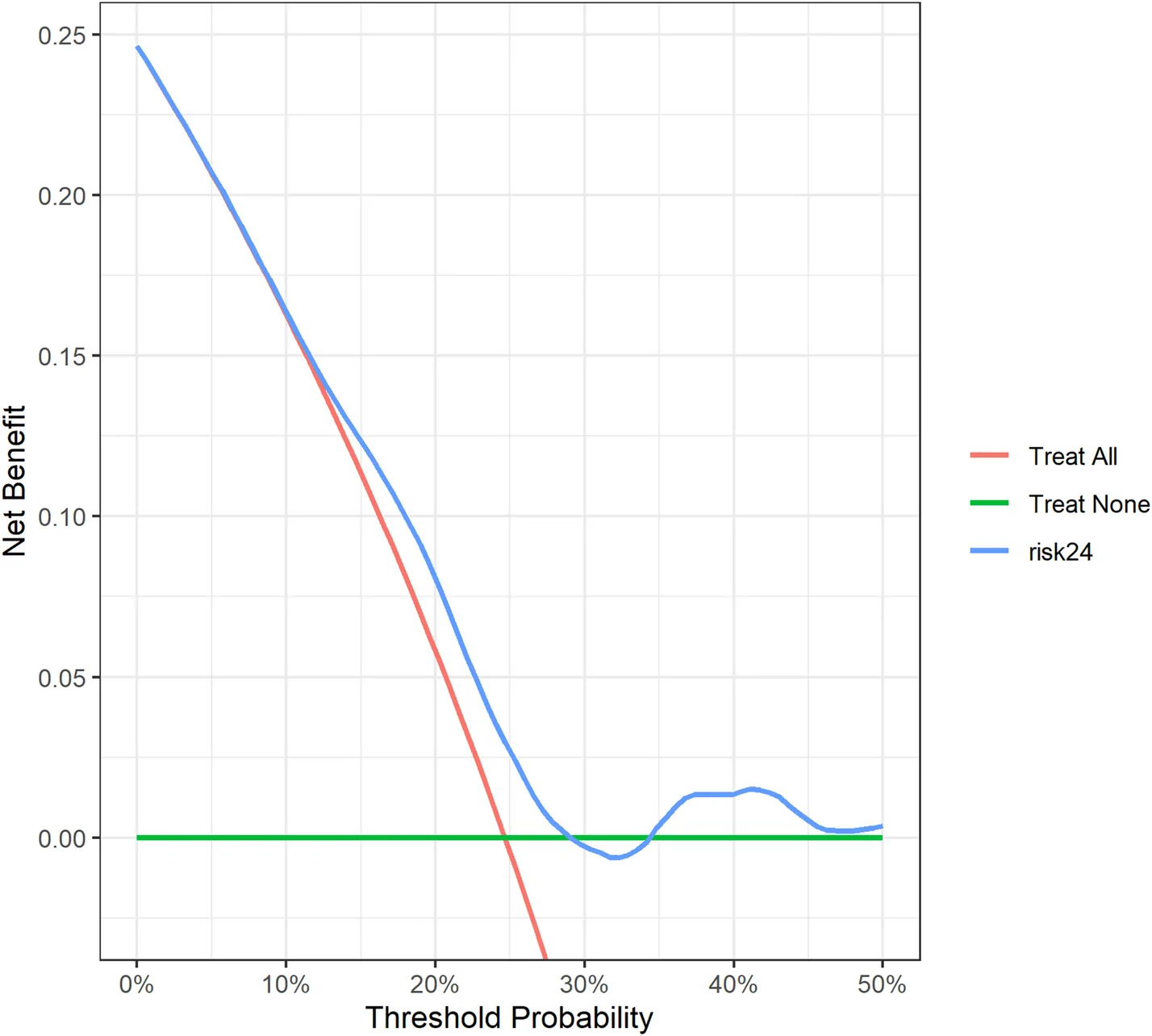
Decision curve analysis (DCA) at 24 months. The DCA curve shows that the model provides positive net clinical benefit across a range of threshold probabilities, suggesting potential utility in clinical decision-making.

Decision curve analysis (DCA) at 24 months demonstrated that the model provided positive net clinical benefit across a range of threshold probabilities (Figure 6), supporting its potential utility in clinical decision-making.

### 3.6 Landmark analysis

To evaluate the temporal stability of HRR's predictive effect, we performed a landmark analysis stratified at 24 months (Table 4). Of the 141 total AVF failure events, 59 occurred within the first 24 months (early phase) and 82 occurred after 24 months (late phase). In the early phase (<24 months), HRR was significantly associated with AVF failure (HR = 1.60, 95% CI: 1.27–2.02, *P* < 0.001). In the late phase (>24 months), the effect estimate remained directionally consistent, although it did not reach statistical significance, likely due to the reduced sample size (HR = 1.47, 95% CI: 0.66–3.29, *P* = 0.347). The formal test for interaction between HRR and time period was not significant (*P* for interaction = 0.766), suggesting that the current data do not provide definitive evidence of a time-dependent effect. However, the wide confidence interval of the late-phase estimate (HR = 1.47, 95% CI: 0.66–3.29) indicates that this analysis was likely underpowered to detect such an effect.

**Table 4:** Landmark Analysis of HRR for AVF failure stratified at 24 months.

Period	N	Events	HR (95% CI)	*P* value
Early failure (<24 months)	279	59	1.60 (1.27–2.02)	< 0.001
Late failure (>24 months)	148	82	1.47 (0.66–3.29)	0.347
*P* for interaction	–	–	–	0.766

Abbreviations: HR, hazard ratio; CI, confidence interval. Note: Early and late phase events sum to the total number of events (59 + 82 = 141).

### 3.7 Sensitivity analysis

To assess the robustness of our findings, multiple imputation was performed as a sensitivity analysis. The imputed analysis yielded consistent results, with HRR remaining independently associated with AVF failure (HR = 1.58, 95% CI: 1.28–1.95, *P* < 0.001) (Supplementary Table S3).The comparison between complete-case and imputed analyses is presented in Supplementary Table S4, confirming the stability of the primary complete-case analysis.

## 4 Discussion

In this retrospective cohort study of 279 patients undergoing first-time AVF creation, we found that preoperative HRR was independently associated with AVF failure after comprehensive adjustment for established risk factors including vessel diameter (HR = 1.65, 95% CI: 1.33–2.05, *P* < 0.001). The model demonstrated moderate discrimination (24-month AUC = 0.608), good calibration, and positive net clinical benefit on decision curve analysis. Notably, a landmark analysis stratified at 24 months revealed that the predictive effect of HRR was directionally consistent in both early and late phases (*P* for interaction = 0.766), although the late-phase estimate was imprecise due to reduced sample size (HR = 1.47, 95% CI: 0.66–3.29). This suggests that the association between preoperative HRR and AVF failure is not limited to the early postoperative period, but the study was underpowered to definitively characterize the temporal pattern of this effect.

It is important to clarify that HRR should be considered a prognostic marker rather than a predictive marker in the context of this study. A prognostic marker provides information about a patient's natural disease course and enables risk stratification, which is precisely what our findings demonstrate: higher preoperative HRR is independently associated with an increased risk of AVF failure, irrespective of any specific intervention. By contrast, a predictive marker would require evidence that HRR identifies patients who differentially benefit from a particular therapeutic or surgical strategy (e.g., intensified surveillance, anti-inflammatory therapy, or alternative surgical techniques). Our observational cohort study was not designed to address this question, as all patients received the same standard-of-care surgical procedure without randomized treatment allocation. Future interventional studies are needed to determine whether HRR can serve as a predictive marker for guiding specific perioperative management strategies.

Our findings extend and complement two recently published studies that explored inflammatory-nutritional markers in AVF outcomes. Zhao et al. (10) developed a nomogram incorporating HRR along with other inflammatory indices to predict AVF maturation failure, while Hu et al. (19) reported an association between the C-reactive protein-to-albumin ratio (CAR) and late AVF dysfunction in a Chinese cohort. However, several important distinctions and gaps should be noted. First, neither study performed a time-stratified analysis to examine the stability of the inflammatory marker's effect across early vs. late postoperative periods. Our landmark analysis addresses this gap by demonstrating that the predictive effect of HRR is temporally consistent (*P* for interaction = 0.766), providing novel evidence that preoperative inflammation-nutrition status exerts a persistent influence on AVF longevity rather than a time-limited effect.Second, while Zhao et al. focused on maturation failure (a predominantly early phenomenon)and Hu et al.used Fine-Gray competing risk models for late dysfunction, our study specifically addresses clinically significant AVF failure (stenosis ≥50% or thrombosis requiring intervention) and provides a comprehensive TRIPOD-compliant model evaluation including calibration and decision curve analysis (DCA), which was not fully addressed in prior reports. Third, our independent validation in a distinct Chinese cohort with a median follow-up of 50 months strengthens the generalizability of HRR as a practical preoperative biomarker.

The steep decline observed in the early phase of the Kaplan-Meier curve warrants careful interpretation. In our cohort, only 7 of 141 failure events (5.0%) occurred within the first 6 months, which is substantially lower than the 20%–50% primary maturation failure rates reported in the literature (4). This suggests that our surgical and perioperative protocols achieved a high rate of successful AVF maturation. The visual steepness of the early curve is likely attributable to the concentration of early events within a narrow time window (2–6 months) and the scale of the y-axis, rather than a high absolute number of early failures. The vast majority of failures (95%) occurred beyond 6 months, consistent with late failure driven by neointimal hyperplasia and stenosis, which supports the use of HRR as a predictor of long-term AVF outcomes.

The biological plausibility of our findings is supported by the dual role of HRR in reflecting both systemic inflammation and nutritional status. Elevated hsCRP indicates ongoing inflammation that promotes endothelial dysfunction, oxidative stress, and neointimal hyperplasia—key processes in AVF stenosis (21). Concurrently, hypoalbuminemia reflects poor nutritional reserve, which may impair vascular healing, reduce the capacity for adaptive remodeling, and compromise the integrity of the vascular wall (22). The combination of these two pathways in a single metric likely explains why HRR outperforms either marker alone, as has been demonstrated in other disease contexts (23, 24). The independent effect of HRR remained significant even after adjusting for vessel diameter, suggesting that the inflammation-nutrition pathway contributes to AVF outcome beyond anatomical factors alone. The lack of a statistically significant association between diabetes and AVF failure in the multivariate model, despite its established role in prior studies, may be explained by the inclusion of HRR, which captures the inflammatory pathway through which diabetes contributes to vascular injury. This suggests that the effect of diabetes on AVF outcome may be at least partially mediated by inflammation.The sustained predictive effect of HRR across both early and late phases, as demonstrated by our landmark analysis, further supports a chronic pathogenic role of systemic inflammation and malnutrition that begins preoperatively and continues to influence vascular remodeling throughout the entire post-surgical course, rather than exerting an effect limited to a specific temporal window.

A legitimate concern regarding the use of a single preoperative HRR measurement is the dynamic nature of its components. CRP is an acute-phase reactant that can fluctuate in response to intercurrent events, and albumin levels may vary with nutritional status. However, several lines of evidence support the prognostic value of a single baseline measurement. First, the HEMO Study demonstrated that while CRP varies considerably over time (within-subject coefficient of variation, CV = 0.878), albumin exhibits substantially less intra-individual variability (CV = 0.061), providing a stable denominator for the HRR calculation (25). Second, a recent cohort study by Hu et al. (19) successfully used a single preoperative C-reactive protein-to-albumin ratio to predict AVF dysfunction over a median follow-up of 36 months, with the highest CAR tertile associated with a 77% increased risk (HR = 1.77, 95% CI: 1.21–2.58) (19). Third, Kuo et al. (26) demonstrated that hemodialysis patients with fluctuated hs-CRP levels had higher vascular access failure rates than those with persistently high hs-CRP, suggesting that even transient inflammatory elevations contribute to adverse outcomes (26). These findings collectively indicate that a preoperative measurement of inflammation-nutrition status captures a baseline risk profile that has sustained prognostic relevance for AVF outcomes.

The clinical implications of our findings are noteworthy. HRR is derived from routine laboratory measurements (hsCRP and albumin) that are already available in most dialysis centers, requiring no additional cost or specialized equipment. This makes it an accessible tool for preoperative risk stratification. Patients identified as high-risk based on elevated HRR could potentially benefit from intensified surveillance, optimized nutritional support, or targeted anti-inflammatory strategies. The positive net benefit demonstrated by DCA further supports the clinical utility of incorporating HRR into decision-making. Moreover, the moderate but consistent AUC across 12, 24, and 36 months suggests that HRR may be particularly useful for identifying patients at risk for long-term AVF failure, which could guide surveillance strategies in clinical practice (27). The directionally consistent effect of HRR across early and late phases, as suggested by our landmark analysis, may carry a clinical implication: preoperative HRR level could potentially inform surveillance intensity beyond the early postoperative period. However, given the imprecision of the late-phase estimate, this finding should be interpreted cautiously and requires validation in larger cohorts. Patients with elevated HRR remain at persistently higher risk throughout the entire lifespan of the AVF, suggesting that routine surveillance should not be relaxed even after successful early maturation.

Our study has several strengths. First, to our knowledge, this is one of the few studies to perform a landmark analysis to examine the temporal pattern of HRR's predictive effect in AVF outcomes, providing novel biological and clinical insights not addressed in prior reports. Second, we adjusted for vessel diameter—a critical anatomical factor—and conducted comprehensive model performance evaluation including time-dependent ROC, calibration curve, and DCA following TRIPOD guidelines, which enhances the clinical interpretability of our findings. Third, we provide complete R analysis code and an anonymized dataset to ensure full reproducibility, facilitating external validation by other centers.

Several limitations merit consideration. First, this was a single-center retrospective study, which limits generalizability and introduces the possibility of unmeasured confounding. Second, the hsCRP missing rate (39.4%) necessitated complete-case analysis as the primary approach; however, it is important to acknowledge that hsCRP was not universally measured but rather ordered at the discretion of treating physicians based on clinical suspicion of inflammation. This non-random missingness introduces the possibility of selection bias, as patients with available hsCRP may have had a higher baseline inflammatory burden, which could influence the observed effect size. Consequently, our findings may not be fully generalizable to unselected real-world populations or to healthcare systems where hsCRP is routinely measured in all AVF candidates. Nevertheless, sensitivity analysis using multiple imputation yielded directionally consistent results, supporting the robustness of our primary findings. Third, we did not incorporate intraoperative technical factors (e.g., anastomotic technique, surgeon experience, or intraoperative heparinization protocols) that may influence AVF outcome. We acknowledge that these factors are important determinants of early AVF maturation, particularly within the first 3–6 months postoperatively. However, our primary endpoint was clinically significant AVF failure (stenosis ≥50% or thrombosis requiring intervention) assessed over a median follow-up of 50 months, and our 24-month landmark analysis captured events well beyond the immediate technical window. Furthermore, evidence suggests that patient-specific inflammatory and nutritional parameters may play a more sustained role in long-term AVF patency compared with surgical variables. Nonetheless, the absence of these surgical covariates represents a limitation, and future studies should adjust for these variables to better isolate the prognostic role of inflammatory-nutritional markers from technical determinants of AVF success. Fourth, detailed data on pharmacological interventions that may influence AVF outcomes—including antiplatelet agents (e.g., aspirin, clopidogrel), anticoagulants (e.g., warfarin, direct oral anticoagulants), and vasodilators (e.g., calcium channel blockers, ACE inhibitors/ARBs)—were not available in our retrospective dataset. Similarly, specific hemodialysis protocols (e.g., frequency, duration, or mode) were not uniformly recorded. We acknowledge these as important limitations. However, the prescribing of these medications is largely driven by underlying comorbidities—including hypertension, diabetes, and cardiovascular disease—which were included as covariates in our multivariate Cox regression model. This adjustment likely accounts for a substantial portion of the confounding that would otherwise be attributed to medication use. Nonetheless, residual confounding cannot be excluded, and future prospective studies should systematically collect medication utilization and dialysis protocol data to better isolate the prognostic role of inflammatory-nutritional markers from the effects of concomitant therapies. Fifth,the model's C-index of 0.594 indicates modest discrimination, suggesting that additional factors—particularly hemodynamic parameters and postoperative flow measurements—may further improve predictive performance. Sixth, the study population was derived from a single center in China, and the results may not be directly generalizable to other populations with different demographic or clinical characteristics. Seventh, the sample size of 279 patients, while adequate for the number of covariates (EPV ≈ 17.6), limits our ability to perform extensive subgroup analyses. Future studies with larger, multi-center cohorts and external validation are needed to confirm our findings. Eighth, our study was designed to evaluate the prognostic value of preoperative HRR for AVF failure, not its predictive value for guiding specific interventions. As an observational cohort study without a comparator group receiving alternative treatments, we cannot determine whether HRR identifies patients who would differentially benefit from specific perioperative strategies. While we demonstrate that HRR is a useful prognostic marker for risk stratification, its role as a predictive marker for treatment selection remains to be investigated in future randomized controlled trials. Ninth, while the outcome assessors were blinded to HRR values at the time of outcome adjudication, it is important to acknowledge that hsCRP and albumin results were part of routine laboratory reports accessible to the treating clinicians. Therefore, complete blinding of the treating team to HRR was not feasible in this retrospective cohort. Additionally, although standardized duplex ultrasound protocols were applied at scheduled follow-up visits, the ultrasound examinations were performed as part of routine clinical care, which may introduce some variability. However, the primary outcome (≥50% stenosis or thrombosis requiring intervention) was based on objective radiological and interventional criteria, which minimizes the risk of detection bias.

Additionally, competing risk from mortality was not applicable in our cohort, as no deaths were observed during follow-up. While this eliminates the immediate concern of competing events in our data, we acknowledge that longer-term follow-up or cohorts with higher baseline mortality risk may encounter this issue, for which Fine-Gray subdistribution hazard models would be appropriate in future studies. The landmark analysis stratified at 24 months was limited by the reduced sample size in the late phase (*n* = 148, with 82 events). The wide confidence interval for the late-phase HRR estimate (HR = 1.47, 95% CI: 0.66–3.29) indicates that this analysis was underpowered to detect a time-dependent effect, even if one existed. Therefore, the non-significant interaction term (*P* = 0.766) should not be interpreted as evidence of a sustained or stable effect, but rather as reflecting the inability of the current data to distinguish early vs. late effects. Future studies with larger sample sizes and longer follow-up are needed to definitively characterize the temporal pattern of HRR's predictive effect.

In conclusion, preoperative HRR is independently associated with AVF failure. The model incorporating HRR demonstrated moderate discrimination, good calibration, and positive net clinical benefit on decision curve analysis, suggesting that HRR may serve as a useful adjunctive tool for preoperative risk stratification. Its simplicity and low cost make it a practical tool for preoperative risk stratification. Prospective multi-center studies are warranted to validate these findings and to evaluate whether HRR-guided interventions can improve AVF outcomes.

## 5 Conclusion

In this retrospective cohort study, we demonstrated that preoperative HRR is independently associated with AVF failure after comprehensive adjustment for established risk factors including vessel diameter (HR = 1.65, 95% CI: 1.33–2.05, *P* < 0.001). The model incorporating HRR demonstrated moderate discrimination (24-month AUC = 0.608), good calibration, and positive net clinical benefit on decision curve analysis. A landmark analysis stratified at 24 months suggested that the predictive effect of HRR was directionally consistent across early and late phases (*P* for interaction = 0.766), although the late-phase estimate was imprecise due to limited sample size. Taken together, these findings support HRR as a simple, widely available prognostic marker for preoperative risk stratification in AVF candidates. Prospective multi-centre studies are warranted to validate these findings and to evaluate whether HRR-guided surveillance or intervention strategies can improve long-term AVF outcomes.

## Data Availability

The datasets presented in this article are not readily available because the anonymized dataset and complete R analysis code used in this study are available in the supplementary materials. The original data cannot be publicly shared due to institutional privacy protection regulations and patient confidentiality requirements. De-identified data are available from the corresponding author upon reasonable request and institutional approval. Requests to access the datasets should be directed to 312855784@qq.com.
