# Supplementary material for: Preoperative high-sensitivity C-reactive protein-to-albumin ratio predicts arteriovenous fistula failure in hemodialysis patients: a retrospective cohort study with landmark analysis
Source: Front Surg. 2026 Sep 9;13:1922562. doi: 10.3389/fsurg.2026.1922562 (PMC13597833; doi:10.3389/fsurg.2026.1922562)
Supplement: Supplementary file 1 [file Table1.docx]

Supplementary Table 1 Missing data patterns in the study cohort

| **Variable** | **Missing_%** |
| --- | --- |
| age | 1.467268623 |
| gender | 0 |
| BMI | 19.751693 |
| smoking | 1.241534989 |
| hypertension | 0 |
| diabetes | 0 |
| HRR | 39.39051919 |
| vessel_diameter | 30.13544018 |
| usage_time | 12.97968397 |
| event | 19.0744921 |
