# Supplementary material for: Preoperative high-sensitivity C-reactive protein-to-albumin ratio predicts arteriovenous fistula failure in hemodialysis patients: a retrospective cohort study with landmark analysis
Source: Front Surg. 2026 Sep 9;13:1922562. doi: 10.3389/fsurg.2026.1922562 (PMC13597833; doi:10.3389/fsurg.2026.1922562)
Supplement: Supplementary file 2 [file Table2.docx]

Supplementary Table 2	Risk table for KM curve

| **ime** | **High HRR** | **Low HRR** |
| --- | --- | --- |
| 0 | 140 | 139 |
| 6 | 136 | 133 |
| 12 | 116 | 127 |
| 18 | 95 | 118 |
| 24 | 68 | 107 |
| 30 | 43 | 89 |
| 36 | 34 | 76 |
| 42 | 29 | 62 |
| 48 | 20 | 52 |
| 54 | 17 | 39 |
| 60 | 15 | 32 |
