# Supplementary material for: Preoperative high-sensitivity C-reactive protein-to-albumin ratio predicts arteriovenous fistula failure in hemodialysis patients: a retrospective cohort study with landmark analysis
Source: Front Surg. 2026 Sep 9;13:1922562. doi: 10.3389/fsurg.2026.1922562 (PMC13597833; doi:10.3389/fsurg.2026.1922562)
Supplement: Supplementary file 3 [file Table3.docx]

Supplementary Table 3	Multiple imputation results

| **Variable** | **HR** | **CI_lower** | **CI_upper** | **P** |
| --- | --- | --- | --- | --- |
| HRR | 1.302008936 | 1.060291329 | 1.598831587 | 0.021111594 |
| age | 1.006584345 | 1.003308667 | 1.009870717 | 0.000133624 |
| gender | 1.10127173 | 0.855802152 | 1.417149304 | 0.45708938 |
| BMI | 0.991262658 | 0.963255517 | 1.02008412 | 0.558100892 |
| smoking | 0.751168798 | 0.526265659 | 1.072185795 | 0.130827147 |
| hypertension | 1.206930793 | 0.78074422 | 1.865760773 | 0.398543081 |
| diabetes | 1.315608183 | 0.978216361 | 1.76936817 | 0.080310975 |
| vessel_diameter | 0.230065444 | 0.108339363 | 0.488558422 | 0.001533737 |
