# Supplementary material for: Preoperative high-sensitivity C-reactive protein-to-albumin ratio predicts arteriovenous fistula failure in hemodialysis patients: a retrospective cohort study with landmark analysis
Source: Front Surg. 2026 Sep 9;13:1922562. doi: 10.3389/fsurg.2026.1922562 (PMC13597833; doi:10.3389/fsurg.2026.1922562)
Supplement: Supplementary file 4 [file Table4.docx]

Supplementary Table 4	Complete‑case vs. imputation comparison

| **Method** | **HR_HRR** | **CI_lower** | **CI_upper** | **P_HRR** |
| --- | --- | --- | --- | --- |
| Complete-case | 0.500572373 | 0.283849051 | 0.717295695 | 5.98304E-06 |
| Multiple imputation | 1.302008936 | 1.060291329 | 1.598831587 | 0.021111594 |
